# Impact of personal and environmental factors affecting exclusive breastfeeding practices in the first six months during the COVID-19 pandemic in Thailand: a mixed-methods approach

**DOI:** 10.1186/s13006-022-00515-3

**Published:** 2022-10-17

**Authors:** Sasitara Nuampa, Ameporn Ratinthorn, Crystal L. Patil, Kornkanok Kuesakul, Sudhathai Prasong, Metpapha Sudphet

**Affiliations:** 1 Department of Obstetrics and Gynecological Nursing, Faculty of Nursing, Mahidol University, Bangkok, Thailand; 2 Department of Human Development Nursing Science, College of Nursing, University of Illinois Chicago, Chicago, IL USA; 3 Department of Obstetrics and Gynaecology, Siriraj Hospital, Mahidol University, Bangkok, Thailand

**Keywords:** COVID-19, Exclusive breastfeeding, Factors, Mixed method, Thailand

## Abstract

**Background:**

Exclusive breastfeeding (EBF) for six months is recommended as one of the most important ways to support child health and survival, particularly during the COVID-19 pandemic. However, breastfeeding women encountered several obstacles during the pandemic. The purpose of this study was to conduct a survey to document breastfeeding practices, EBF rates and associated factors with six month exclusive breastfeeding during the second wave of the coronavirus outbreak in Thailand.

**Methods:**

A mixed-methods design that included a cross-sectional survey (*n* = 390) and semi-structured in-depth interviews (*n* = 15) was carried out between August and November 2021. Participants were women aged ≥ 15 years who had given birth within 6–12 months before data collection and delivered in three public hospitals in the top three provinces with the most severe COVID-19 outbreaks.

**Results:**

The median duration of EBF was four months (interquartile range, IQR: 1–6 months) and 37.4% of women exclusively breastfed for six months. From binary logistic regression models, several personal factors were associated with exclusive breastfeeding for six months including being a housewife (AOR 2.848; 95% CI 1.512, 5.367), perceived sufficiency of family income (AOR 2.502; 95% CI 1.362, 4.594), working from home/business (AOR 2.071; 95% CI 1.074, 3.995), breastfeeding intention (AOR 1.162; 95% CI 1.116, 1.210), and maternal age (AOR 0.932; 95% CI 0.882, 0.986). From qualitative interviews, women who were able to exclusively breastfed during the outbreak explained five themes that were a protective shield; I have to save money, I could spend all my time with my baby and breastfeed, spousal support is valuable, and opportunity to avoid the obstructed beliefs about exclusive breastfeeding.

**Conclusions:**

Mothers with higher socioeconomic status and who were unemployed/worked from home and had support structures in place were able to successfully EBF during the COVID-19 outbreak. Healthcare providers can better support breastfeeding if they provide informational support and allow family members to participate in breastfeeding programs, especially spouses who provided key emotional and tangible support during pandemic.

## Background

COVID-19 now in its third year, has disrupted lives and livelihoods and caused at least 6.3 million deaths worldwide [1]. In Thailand there were 4.4 million reported cases of infection and 30,022 deaths, primarily with the Delta variant during the second wave of COVID-19 [2]. Thailand implemented social measures in April 2020, including a full-scale national lockdown, curfews, and a 14-day mandatory quarantine for international travelers. Economic activities were resumed on May 1, 2020, until the end of the year in the first wave of COVID-19. On December 17, 2020, a second wave and the total lockdown led to a serious negative economic impact [2].

Despite increasing uptake of the COVID-19 vaccine globally, newborns and young children remain vulnerable since they are not able to receive this new vaccine [3]. Breast milk is essentially the baby's first vaccine and is an important and impactful way to support child health and survival, especially during the COVID-19 pandemic [4, 5]. Both a COVID-19 infection and immunization may result in the production of substantial antibodies in human milk [6–8]. As a result, optimal breastfeeding should be promoted globally during the COVID-19 outbreak to protect the health of infants and young children. This aligns with WHO recommendations for mothers worldwide to exclusively breastfeed infants for the first six months of life to achieve optimal growth, development, and health. After the first six months, children should be provided with nutritious complementary foods and continue to nurse until the age of two years or older [9].

Throughout the pandemic, breastfeeding women have struggled to gain support and encountered several obstacles causing them to discontinue breastfeeding before they were ready [10–13]. Several personal and environmental factors have interacted to influence breastfeeding outcomes during the COVID-19 pandemic. Lockdowns, and the associated isolation, stress, and distress, have had a negative psychological impact on breastfeeding mothers [14, 15]. Furthermore, non-breastfeeding mothers reported higher fear scores on the COVID-19 Scale COVID-19 (FCV-19S) suggesting that fears may be associated with early weaning [16, 17]. Vaccination acceptability and an accurate understanding of its safety may also be a key factor in infant feeding decisions [18].

Social support, a well-established environmental factor, is important to successful breastfeeding practices [10, 19, 20]. However, in the context of COVID-related mitigation strategies and policies, in-person lactation support was limited and mothers may not have received the needed support. Left unaddressed, this nonexistent or diminished support could negatively impact breastfeeding initiation and duration [13]. Hence, alternative support structures and resources are needed when the typical face-to-face health service support is not available [14].

Thailand is the lowest-ranking country in the Asia/Pacific region for exclusive breastfeeding (EBF) [21] and is far from reaching the global target of fifty percent [9]. In just three years, between 2016 and 2019, EBF dropped by 50%, decreasing from 23.1% to fourteen percent [22]. The COVID-19 pandemic has affected household income due to lay-offs, furloughs, reduced work hours and has disrupted social service delivery. The impact of COVID-19 on women working in the informal sector has been especially harsh and likely affected infant feeding practices and exclusive breastfeeding [23].

Prior to the pandemic, poor EBF rates were associated with cultural and structural constraints [24]. Culturally, mothers expressed embarrassment about breastfeeding or expressing milk in public. Sometimes grandparents served as an obstacle to EBF because as respected caregivers they encouraged adding other sustenance, such as water, rice, and fruit, to the infant diet [24]. Mothers working outside the home had difficulty maintaining lactation because of the challenges and stress of combining breastfeeding with work demands [24, 25]. Given the new context of the COVID-19 pandemic, the purpose of this study was to examine associations between the personal and environmental factors and exclusive breastfeeding.

## Methods

### Aims

To examine the factors associated with exclusive breastfeeding practices in the first six months and to explore what allowed some women to EBF for a longer duration during the COVID-19 pandemic in Thailand.

### Design

This study, conducted in Thailand, was part of Breastfeeding Behaviors and Experiences among Postpartum Mothers Encountering the Pandemic Situation of Coronavirus 2019. The mixed-methods design followed an explanatory sequential approach [26]. First, a cross-sectional survey was completed to examine the factors influencing exclusive breastfeeding practices during the second wave of the COVID-19 pandemic. This was followed by a qualitative study in which in-depth interviews were conducted to explore the experiences and perspectives of women who were able to exclusively breastfeed during this crisis.

### Setting

Three tertiary government hospitals from the three provinces with the highest rates of COVID-19 infection during the second wave in Thailand from December 2020 to February 2021 [27], were randomly selected for inclusion in this study that took place between August and November 2021 [27]. The protocol was approved by the Ethics Committee of three hospitals.

### Sample

Participants were women aged ≥ 15 years who had given birth within 6–12 months before data collection and delivered at one of these three public hospitals were eligible to participate. A sample size of 390 mothers was calculated by using the breastfeeding proportion at six months in Thailand of 0.41 [22] with a 95% confidence interval and precision (d) of 0.05 and a 5% attrition rate. To calculate the number of participants and childbirth rates per month, data from a representative tertiary level government hospital in each province was used [28].

A convenience sample was used to recruit participants. We first identified potential participants from postpartum medical records and then contacted them via telephone to explain the study and invite them to participate. Inclusion criteria included women aged ≥ 15 years who were able to read Thai, had a smartphone, did not have a preterm birth (gestational age ≥ 37 weeks), and had no documented history of COVID-19 infection.

All women received an explanation of the study procedures, and those who agreed to participate signed an electronic informed consent form. The women took the survey using online questionnaire sent to their smartphones. After survey completion, mothers (*n* = 15) who had exclusively breastfed for six months were identified and recruited to participate in-depth semi-structured interviews.

### Data collection

Once consented, women (*n* = 390) were sent the three links to an online cross-sectional survey and asked to complete them within 24 h; those who did not complete it were excluded (*n* = 22, 5.6%). However, we collected the data until complete optimal cases. From the survey data, women who exclusively breastfed for six months were identified and asked to participate in a 45–60 min qualitative interview. Semi-structured in-depth interviews were conducted by a video call to explore perceptions and experiences related to exclusive breastfeeding. Questions about attitude toward breastfeeding, family and health provider social support during the COVID-19 situation were included. Field notes and an interview codebook were used for the analysis of the semi-structured interviews.

### Measures

#### Survey

A self-administered online survey that consisted of three sections was sent to participants through three different links consisted of eight questionnaires (Table 1). The definition of exclusive breastfeeding (EBF) in this study was defined providing only breast milk from the mother or wet nurse for the first six months and no other solids or liquids, except for drops or syrups consisting of vitamins, minerals, supplements, or medicines [29].

**Table 1:** List of self-administered online questionnaires

Outcome	Specific measure	Cronbach’s alpha, this study
**Section 1**		
Sociodemographic	Maternal age (years), educational status, monthly family income, income status, work status, family characteristics, maternal leave	N/A
Reproductive and infant feeding history	parity, pregnancy planning, maternal complications, mode of delivery, infant age (month), breastfeeding intention, previous breastfeeding experience, influential people on breastfeeding practices, infant-feeding patterns, and reasons stopped breastfeeding	N/A
**Section 2**		
Fear of COVID-19 Scale [16]	7 items, 5-point Likert scales ranging from 1 (strongly disagree) to 5 (strongly agree), range 7–35, higher scores indicating a high level of fear. Demonstrated reliability and validity [16, 30]	0.83
Knowledge, Attitudes, and Practices (KAP) Towards COVID-19 Scale [31]	12 items (4 aspects: clinical manifestations, transmission routes, prevention, and control of COVID-19), a true-or-false basis with an additional "I don't know" option, range 0–12, higher score denoting a better knowledge of COVID-19. Demonstrated reliability and validity [31]	0.70
The State Anxiety Inventory Form Y-1 (STAI-S) [32]	20 items, 4-point Likert scales ranging from 1 (almost never) to 4 (almost always), range 20–80, a higher score denoting a high anxiety level. Demonstrated reliability and validity [33]	0.93
**Section 3**		
COVID-19 Vaccination Acceptability Tools [34]	**Part 1:** 27 items (24 items about a possible COVID-19 vaccination and added 3 items on BF-related COVID-19), eleven-point scale (0–10) from "strongly disagree" to "strongly agree", range 0–270, higher scores denoting a more positive attitude **Part 2:** 1 item of vaccination intention, eleven-point scale (0–10) from "very unlikely" to "very likely", range 0–10, higher scores denoting higher intention for COVID-19 vaccination Demonstrated reliability and validity [34]	0.82
The Exclusive Breastfeeding Social Support Scale (EBFSS scale) [35]	16 items (assessed 3-domains of family support; emotional, informational, and instrumental support), 3-point Likert scales, ranging from 1 (no help at all), 2 (less than you would like), and 3 (as much as you would like), range 16–48, a higher score indicating stronger support for EBF. Demonstrated reliability and validity [35]	0.90
The Health Provider Support in Breastfeeding during COVID-19 (HSBF-COVID scale) [13, 36–39]	15 items (assessed 4-domains of healthcare support; emotional, informational, instrumental, and appraisal support), 3-point Likert scales ranging from 1 (no help at all), 2 (less than you would like), and 3 (as much as you would like), range 15–45, higher scores indicating a higher level of health provider support for BF during pandemic	0.92

#### Interviews

A 16-question semi-structured interview guide was used to elicit infant-feeding experiences during the coronavirus outbreak, mothers’ perceptions about this social crisis, and the support they received from family and health providers that facilitated their capacity to exclusively breastfeed.

## Instrument translation and validity

A translation of the instruments from the English into the Thai was conducted to ensure content, semantic, and technical equivalence were managed. Back translation of the instruments was conducted to ensure that the original (English) and second language (Thai) versions of the instruments measured the same words and concepts [40]. The questionnaires and interview guide were evaluated for content by experts, including obstetricians, nurse professors of obstetrics and gynecology, and infection control, while Cronbach's alpha and Kuder-Richardson (KR-20) were used to show reliability of six instruments. The interview guide was developed by qualitative research experts and validated by three experts in breastfeeding and COVID infection to ensure accurate concepts and clear and accurate wording [41].

### Data analysis

#### Survey

The data analysis was performed using the SPSS 28.0 software. Descriptive statistics were used for population characteristics; chi-square, Fisher's exact test, t-test, Mann–Whitney U test and confidence interval were used to report differences between groups, and the significance level was *p* < 0.05. Logistic regression models were estimated to identify the odds ratio of EBF at six months postpartum. Through enter command, the factors associated with EBF at six months were identified. The adjustment variables in the final models were marital status, work characteristics, income status, breastfeeding experience, breastfeeding intention, knowledge about COVID-19, anxiety state, fear of COVID-19, attitude toward COVID vaccination, intention to receive COVID vaccination, family support during COVID-19, and provider support during COVID-19.

#### Interviews

Verbatim transcriptions of the recorded interviews were analyzed using content analysis [42]. Field notes, taken immediately after the interviews, helped document early awareness of important topics. Transcripts were read line-by-line and coded to identify key concepts. Small clusters of codes were aggregated into more meaningful categories, followed by sorting various categories into themes. Data collection and analysis were conducted concurrently to ensure that new concepts emerging from the interviews could be explored in detail in the remaining interviews [41, 42].

## Results

### Survey

On average, women were 30.59 (± 5.69) years of age. Nearly half worked outside the home, with about 20% working from home or owning their own business. About 60% of the mothers reported their family income to be sufficient. The majority, 83% did not have a history of perinatal or postpartum complications (Table 2). Infants were on average 7.86 (± 1.50) months old with a mean infant birthweight of 3,070 g (± 424 g), and nearly all of infants were considered healthy (*n* = 382, 97.9%). To compare to the factors associated with those who did not exclusively breastfeed for six months to those who did, we first completed bivariate analyses. These two groups significantly differed on four sociodemographic factors and two breastfeeding factors (Table 2) and three environmental and psychological factors (Table 3).

**Table 2:** Socio demographic, childbirth, and breastfeeding data and bivariate analysis comparing factors based on duration of EBF

Characteristics	Total (*N* = 390)		Group		*p*
			**EBF < 6 months (*n* = 244)**	**EBF 6 months (*n* = 146)**	
**Socio demographic data**					
Maternal age (years)	30.59 ± 5.69		30.50 ± 5.93	30.74 ± 5.29	0.712
**Marital status**					
Single	42 (10.8)	35 (14.3)		7 (4.8)	0.013*
Married	334 (85.6)	201 (82.4)		133 (91.1)	
Separated/Divorced	14 (3.6)	8 (3.3)		6 (4.1)	
**Educational status**					
Primary school	12 (3.1)	10 (4.1)		2 (1.4)	0.021*
Secondary school	180 (46.2)	121 (49.6)		59 (40.4)	
Bachelor degree	168 (43.1)	96 (39.3)		72 (49.3)	
≥ Master degree	30 (7.7)	17 (7.0)		13 (8.9)	
**Work characteristics**					
Housewife	128 (32.8)		67 (27.5)	61 (41.8)	0.001*
Work from Home	82 (21.0)		47 (19.3)	35 (24.0)	
Outside Work	180 (46.2)		130 (53.3)	50 (34.2)	
**Income status**					
Sufficient	230 (59.0)		126 (51.6)	104 (71.2)	0.000*
Insufficient	160 (41.0)		118 (48.4)	42 (28.8)	
**Pregnancy and childbirth data**					
**Parity**					
Primiparous	184 (47.2)		118 (48.4)	66 (45.2)	0.546
Multiparous	206 (52.8)		126 (51.6)	80 (54.8)	
**Mode of delivery**					
Vaginal delivery	177 (45.4)		119 (48.8)	58 (39.7)	0.083
Caesarean section	213 (54.6)		125 (51.2)	88 (60.3)	
**Breastfeeding data**					
Breastfeeding intention (*n* = 383)					
Yes	372 (97.1)		227 (59.3)	145 (100.0)	0.005*
No	11 (2.9)		11 (2.9)	0 (0.0)	
Breastfeeding experiences					
Yes	164 (42.1)		92 (37.7)	72 (49.3)	0.025*
No	226 (57.9)		152 (62.3)	74 (50.7)	

^*^p-value < 0.05

Data are shown as *n* (%) and mean ± standard deviation. Chi-squared testing was used for categorical variables (marital status, work characteristics, income status, parity, mode of delivery, and breastfeeding experience). Fisher’s exact test was used for categorical variables and violate chi-squared assumption (educational status and breastfeeding intention). Z-score in Mann–Whitney U test for continuous variables with not normally distributed data

**Table 3:** Psychological and environmental data and bivariate analysis comparing factors based on duration of EBF

Characteristics	Total (*N* = 390)	Group		*p*
		**EBF < 6 months (*n* = 244)**	**EBF 6 months (*n* = 146)**	
**Psychological data**				
Attitude toward vaccination	175.56 ± 32.49	177.53 ± 33.84	172.28 ± 29.92	0.120
Intention to vaccinate	8.71 ± 2.23	8.62 ± 2.24	8.84 ± 2.22	0.072
Anxiety about COVID-19 situation	51.85 ± 10.60	51.06 ± 10.64	53.18 ± 10.41	0.036*
Fear about COVID-19	25.45 ± 4.99	25.70 ± 5.15	25.03 ± 4.68	0.211
COVID-19 knowledge	9.57 ± 1.26	9.55 ± 9.61	9.61 ± 1.33	0.348
**Environmental data**				
Family type				
Nuclear	233 (59.7)	149 (61.1)	84 (57.5)	0.491
Extended	157 (40.3)	95 (38.9)	62 (42.5)	
Family support	43.26 ± 5.78	42.82 ± 6.00	43.96 ± 5.31	0.031*
Provider support	40.25 ± 6.13	39.84 ± 6.31	40.93 ± 5.78	0.028*

^*^p-value < 0.05

Data are shown as *n* (%) and mean ± standard deviation. Chi-squared testing was used for categorical variables (family type). Z-score in Mann–Whitney U test for continuous variables with not normally distributed data

### Breastfeeding practices

Almost all the mothers reported that they intended to breastfeed (95.4%) and 37.4% exclusively breastfed for six months. The average duration of breastfeeding intention was 11.73 months (± 7.55) with a range between 1–36 months and the median duration of EBF was four months (interquartile range, IQR: 1–6 months). Nearly half of the mothers had breastfed before (42.1%).

### Association between EBF for 6 months and Covid-19-related personal and environment factors

Binary logistic regression was used to assess personal and environmental factors related to six months of exclusive breastfeeding during the COVID-19 pandemic (Table 4). The coefficient and adjusted odds ratio (AOR) of the multivariable binary logistic regression model demonstrated that personal factors were significantly associated with EBF for six months during the COVID-19 pandemic. Increasing maternal age was associated with a reduced likelihood of exclusive breastfeeding for six months [AOR 0.932; 95% CI 0.882, 0.986]. Mothers who were unemployed [AOR 2.848; 95% CI 1.512, 5.367] and worked from a home or private business [AOR 2.071; 95% CI 1.074, 3.995] were more likely to exclusive breastfeed for six months. Women who felt their family income was sufficient were also more likely to exclusive breastfeed for six months [AOR 2.502; 95% CI 1.362, 4.594]. Intention, those who planned to breastfeed for a longer period were also more likely to exclusive breastfeed for six months [AOR 1.162; 95% CI 1.116, 1.210].

**Table 4:** Effect of personal and environmental factors on exclusive breastfeeding for the first 6 months of an infant’s life during the COVID-19 pandemic in Thailand

Characteristics	Unadjusted odds ratio	95%CI of OR	*p* -value	Adjusted odds ratio	95%CI of OR	*p* -value
**Maternal age**	1.007	0.971, 1.044	0.697	0.932	0.882, 0.986	0.013*
**Marital status**						
Single	0.267	0.070, 1.012	0.052	0.285	0.059, 1.361	0.116
Married	0.882	0.299, 2.600	0.820	0.787	0.214, 2.897	0.719
Separated/Divorced	-	-	-	-	-	-
**Educational status**						
Primary school	0.262	0.049, 1.405	0.103	0.223	0.028, 1.754	0.154
Secondary school	0.638	0.290, 1.400	0.262	0.454	0.157, 1.312	0.145
Bachelor degree	0.981	0.448, 2.148	0.961	0.780	0.302, 2.011	0.607
≥ Master degree	-	-	-	-	-	-
**Work characteristics**						
Housewife	2.367	1.470, 3.811	0.000*	2.848	1.512, 5.367	0.001*
Work from home/Private	1.936	1.122, 3.342	0.018*	2.071	1.074, 3.995	0.030*
Business	-	-	-	-	-	-
Outside work						
**Perceived income status**						
Sufficient	2.319	1.497, 3.592	0.000*	2.502	1.362, 4.594	0.003*
Insufficient	-	-	-	-	-	-
**Breastfeeding experience**						
No	0.622	0.411, 0.942	0.025*	0.716	0.421, 1.219	0.219
Yes	-	-	-	-	-	-
**Intended breastfeeding duration**	1.158	1.118, 1.200	0.000*	1.162	1.116, 1.210	0.000*
**Knowledge about COVID-19**	1.042	0.883, 1.230	0.625	0.899	0.730, 1.107	0.315
**Anxiety about COVID-19 situation**	1.019	0.999, 1.040	0.057	1.000	0.972, 1.030	0.988
**Fear about COVID-19**	0.973	0.934, 1.014	0.197	1.002	0.940, 1.068	0.958
**Attitude toward COVID-19 vaccination**	0.995	0.989, 1.001	0.123	0.992	0.983, 1.001	0.096
**Intention to receive COVID-19 vaccination**	1.047	0.952, 1.151	0.348	1.040	0.923, 1.172	0.517
**Family support during COVID-19**	1.037	0.998, 1.077	0.060	1.041	0.989, 1.096	0.125
**Provider support during COVID-19**	1.031	0.995, 1.068	0.093	1.022	0.974, 1.072	0.374

Overall percentage 74.6; Hosmer and Lemeshow Test (*p* = 0.288); Nagelkerke R^2^ 40% Dependent variables; Achieving 6-month EBF (yes = 1, a reference case), less than 6-month EBF (no = 0, a non-reference case)

^*^p-value < 0.05

## Qualitative Interviews

### Perceptions of long-term EBF mothers

In Thailand the second waves of COVID-19 started the end of December 2020. Everyone, including participants in this study, was affected by national mitigation strategies that included limited time in public spaces, school closures, working from home, and no transportation in some provinces. While participants accepted and understood rationale behind these protective measures, their lifestyles changed, they felt isolated, and there were negative implications for family income due to job loss and hour reductions. Some mothers reported that the COVID-19 situation did not impact their postpartum experiences because the timing aligned with Thai postpartum customs of staying home to heal, bond, and protect illness. Others, however, described how COVID-19 impacted on their postpartum and breastfeeding experiences.

Fifteen mothers who were able to exclusively breastfeed for six months were asked to talk about their motivations and experiences with social support while mitigation strategies were in place. They described personal and environmental dimensions including the role of a positive attitude toward breastfeeding, flexible work schedules, and family support on their experience.

From these in-depth conversational style interviews, five themes were identified: *A protective shield* describes applying the information and recommendations for breastfeeding to the pandemic situation and recognition of its potential for immunological protection. *I have to save money* reflected the economic impacts of the pandemic and the recognition that reduced income and cost of formula could be somewhat averted with breastfeeding. *I could spend all my time with my baby and breastfeed* captured experiences related to limited mobility and changes to work schedule. Women noted having the time to breastfeed. The *spousal support is valuable* theme illustrates the importance of spousal support. The themes, *opportunity to avoid the obstructed beliefs about EBF*, illustrates that intergenerational conflicts about how to feed infants were less of an issue when other family members could not stay during the postpartum period due to pandemic restrictions.

### A protective shield

Women described how healthcare providers and social media positively impacted their decisions to exclusively breastfeed. They described hearing messages about the advantages that breastfeeding had for improving babies’ immunity systems, which was especially valued in the context of an airborne infectious disease pandemic. Most did not connect breastfeeding to the production of COVID-19 antibodies and transfer via human milk from infection or vaccines, although only a few did. The mothers simply absorbed and recognized that breastfeeding was best way to provide the potential immunity for their infants. The possibility of protection motivated them to commit to exclusive breastfeeding.

A 25-year-old multiparous mother with an 8-month-old infant and was continuing to breastfeed at the time of the interview explained that she had intended to EBF for six months because she would like to have a healthy infant and the pandemic only increased her drive. Even though she experienced several breastfeeding issues (flat nipple, poor attachment, and sore/cracked nipple), she successfully used a breast pump to ensure that the baby would get breast milk. She explained:

*“I did it on account of my baby. I thought he would surely get immunity he could never get from any milk product if he had only my breast milk, so that is what I strongly intended. I made every effort I could, despite the fact that I had many obstacles, as I previously stated . . . And during this COVID outbreak, I wanted to provide him with increasing immunity, so I tried even harder.” (ID16, L448)*

Another mother who described having positive breastfeeding experiences with her older, healthy daughter believed that six months of EBF was best for her new baby’s health. She used a shield analogy to explain her though process, she said.

*“The doctor recommended that I provide exclusive breastfeeding for 6 months. That is the best way to protect my baby. Could it be possible for me to transfer the best thing and create a protective shield with virus protection of my own? It is true; my older daughter never gets sick. It gives me confidence in my breast milk.” (ID4, L134)*

A primiparous mother with a positive attitude toward breastfeeding described breastfeeding as the best food for her baby. In the COVID-19 situation, she said that it was her sound intention to breastfeed optimally to prevent the coronavirus. She said:

*"He is my first child, and I waited nearly ten years for him. I want to give him the best thing that I can. I know breastfeeding is best for his health, particularly during this COVID-19 pandemic that’s attacking us like this." (ID28, L232)*

Mothers working outside of the home were concerned about exposing their family and the infant to COVID-19. In response, several highlighted benefits of breast milk to their baby’s health. One 33-year-old mother with two children said that she decided to pump and store breast milk after returning to work. She explained:

*" I want to breastfeed as long as I can. It will provide strong immunity when she grows up. I believe in the quality of breast milk; everyone talks about the same thing on social media. But it wasn’t safe to breastfeed my baby directly after returning to work. So, I’ve changed from breasts to a bottle. It makes me happier." (ID9, L264)*

#### I have to save money

All of the mothers admitted that the COVID-19 situation had negative effect on their income. A few mothers noted that breastfeeding was cheaper than purchasing formula, so it was a way save money for their families and a major reason to continue exclusive breastfeeding. There were hidden costs associated with the pandemic. Families had to purchase items such as masks and alcohol hand gel, at the same time their income was decreasing.

A primiparous mother who exclusively breastfed for six months and who had returned to work after three months said that she had not planned to exclusive breastfeeding. She did recognize the economic benefits, she explained:

*"Actually, I did not plan anything for this. I think the main reason to continue breastfeeding is that I did not have enough money to buy formula milk. It’s so expensive. Nowadays, I have to save as much as I can on family cost." (ID11, L145)*

Another first-time mother described how she did not want to spend money on formula milk since the pandemic left her spouse unemployed. She lamented:

*"During the COVID-19 pandemic, my family was badly impacted. My husband had to stop working because he is a fitness trainer. Above all else, I have to save money. If I do not have income and I have to buy formula milk, I think I won’t be able to handle the situation, because my baby eats a lot." (ID 21, L278)*

#### I could spend all my time with my baby and breastfeed

During the second of COVID-19, travel and social group size mitigation strategies impacted to the use of public transportation. Several mothers also experienced work-related impacts. Some mothers quit their jobs during the postpartum period because they worried about infection. Others took advantage of workplace policies related to maternity leave and working from home. In some ways, these mitigation strategies positively affected breastfeeding practices because mothers noting they could easily and conveniently breastfeed. For example,

A mother with two children described feeling lucky to have the opportunity to work from home. The office work from home policy allowed her the opportunity to work and breastfeed safely. She explained:

*"I think COVID-19 had some positive aspects in terms of mothers who could work from home. If I had to return to work, it would be difficult to pump breast milk and store it in the workplace, because I wouldn’t know how to clean for a virus-free environment. I might not be sure whether to give that breast milk to my baby. I think it would have been so hard to give my baby only breast milk in that situation." (ID 22, L246)*

#### Spousal support is valuable

Several mothers described the various ways the that mitigation strategies affected their spouse's career. Some spouses worked from home, others worked less, and still others were furloughed, paused, or lost their jobs. While there for financial implications, many mothers also saw this as a good opportunity for providing socials support when other family members could not. The support they described included food preparation, housework, taking care of the baby, and encouraging breastfeeding. They also admitted that some of the spousal support was motivated by the desire to save money along with better health of the baby. For example, a mother with one child explained that the isolation of a nuclear family was stressful. She described that when her spouse was home due to COVID, there were some advantages. She explained,

*"During the early postpartum period, my husband was still working outside, as usual, and I tried to take care of my baby alone like a full-time mother. I was so stressed, sleepless, fatigued, and crying alone. When COVID infections increased, it let to many places' closings temporarily, and my husband’s workplace had to close as well. Since then, I’ve had wonderful support from my husband. He helps me a lot, such bathing for the baby, playing with the baby, and lulling the baby to sleep. It makes me so happy and convenient." (ID 21, L87-94)*

Another first-time mother and her spouse decided it was best for her quit her job. They reasoned that this was the best way to keep both mother and infant safe from infection when the rates were high, particularly in the Bangkok metropolitan area where they lived. She explained:

*"My husband told me that the COVID situation at that time was seriously higher than the infected cases at three months postpartum. So, he told me to quit my job and stay home to take care of our baby only. He would take care of us and his family during the difficult time. I could spend all my time with my baby and breastfeed her continuously." (ID7, L232)*

#### Opportunity to avoid the obstructed beliefs about EBF

Several mothers highlighted that social distancing reduced the influence of grandmothers who often cause the early introduction of water and solid foods. One mother described a difficult situation that happened while her grandmother was there to support her after the birth of the baby. They ended up having arguments about their contradictory infant feeding beliefs. She felt that COVID-19, social distancing, and less visiting from extended family members, gave her the opportunity to feed her baby the way she wanted and supported her ability to exclusively breastfeed for six months. She said:

*"I had a problem about breastfeeding with my grandmother. She had strong traditional beliefs about early introduction of water and mashed bananas at three months. During the early days of COVID, she stayed with us about two weeks. She blamed me a lot for not providing baby foods. I cried every time she blamed me. I stood my ground and prohibited her from adding other foods. Fortunately, the COVID-19 pandemic increased her serious situation, so she had to return home." (ID 4, L152)*

## Discussion

Results showed that 37.4 percent of participants in the survey were able to exclusively breastfeed for six months. Thai women who unemployed, had sufficient income, worked from home or owned private businesses, and intended to breastfeed for a longer duration were more likely to exclusively breastfeeding for six months during the COVID-19 pandemic, while increasing maternal age reduced the likelihood. The association with increased maternal age contradicts findings reported previously. Whipps (2017) reported that lower maternal age was associated with a shorter duration of exclusive breastfeeding [43]. Adolescent mothers due to a complex combination of factors, including family conflict, are also less likely to EBF to six months [20]. More research is warranted to understand other factors affecting older mothers during COVID-19.

Regardless of other factors, breastfeeding intention is a powerful factor in breastfeeding behaviors [44, 45]. A study conducted in the Congo, showed that mothers who did not plan to exclusively breastfeed had a higher risk of discontinuation before six months [46]. Our study reinforced this general knowledge and showed that even during the COVID-19 pandemic, intention mattered. Women who intended to exclusively breastfeed were 1.2 times more likely to be doing so at six months compared to mothers that intended to breastfeed for shorter durations.

The World Bank Group (2020) described the collapse of global economic activity due to the COVID-19 as the deepest recession since World War II [47]. The EBF rate of low-income mothers from Southern California dropped from 15.66% before COVID-19 to 10.38% after the outbreak [48]. Prior to the pandemic higher income increased the likelihood of continued breastfeeding by 1.24 times among United States (US) women [49]. January 12, 2020 marked the beginning of the pandemic for Thailand. Over these 2.4 two years there has been increased economic disruption and social problems. Incomes were affected. The 41% of Thai women who reported insufficient family income more than twice as likely to discontinue exclusive breastfeeding compared to those with sufficient family income. During the COVID-19 pandemic, mothers in difficult economic situations may abandon breastfeeding to look for employment to increase the household income may be increasingly difficult for mothers living in families with low socioeconomic status.

Employment is often cited as an obstacle to exclusive breastfeeding [24, 50, 51]. Like results from a survey Brazil, working mothers in this study were 1.73 times more likely to discontinue exclusive breastfeeding compared to unemployed mothers [50]. With COVID-19, the female unemployment rate in Thailand rose by 1.1% in 2019 and 4.5% in 2020 [23]. Like the qualitative result of this study, a US-based qualitative study also showed that breastfeeding mothers felt that the pandemic had positively influenced their breastfeeding journeys because it allowed for extended maternity leave. However, there could be short and long-term economic stress related to income loss which could then lead to possible increases household power differentials. These same mothers were concerned about safely expressing breast milk on their return to work [13]. This study also showed that most women had a positive attitude toward breastfeeding and that working mothers were worried about transmitting the virus while pumping and storing breastmilk at work. Women make up 44% of the informal sector workforce. The impact of the pandemic on the informal sector was especially harsh. These women work in the service industry with jobs that were most vulnerable during lockdown. In the informal sector, women have the least social protection because this work is not covered under social security schemes [23]. For them, this uncertainty likely affected their decisions to offer formula feeding or wean from breastfeeding during the pandemic. Type of work and income loss certainly affected breastfeeding practices. Conversely, for working women that had workplace policies allowing them to work from home, the increased flexibility allowed for more attention to infant caretaking and improved infant feeding practices.

Family and paternal support are important both physically and emotionally and potentially affect breastfeeding decisions [37]. In this study, social support was not significantly associated with exclusive breastfeeding for six months. These findings are inconsistent with the previous studies that found that lack of healthcare and family support negatively affected breastfeeding practices of Thai mothers after the first COVID-19 lockdown in July–October 2020 [52]. COVID-19 pandemic disrupted lifestyles and social distancing rules changed the way social support looked for postpartum families [14]. According to internal recommendations [4], mothers in this second wave may receive remarkable information about human milk benefits from government campaigns and updated evidences [6, 7]. In contrast during the first wave, an uncertain situation of the COVID-19 pandemic led to misinformation about the safety of breastfeeding and an inability to breastfeed if the mother had symptoms of COVID-19 [53]. Over two years, mothers may adapt their lives to find available resources and support through online platform more than the past [54].

Interview data supported many aspects of the quantitative findings. According to the theory of planned behavior, attitude, subjective norms, and perceived control directly influence intentions to engage in a health behavior [55]. Women’s intended exclusion breastfeeding duration was associated with their actual exclusion breastfeeding duration. Women’s discussions of breastfeeding supported the hypothesis that intention may be influenced by positive attitudes toward breastfeeding associated with the benefits of increased immunity to prevent COVID-19 infection and the cost savings when family income is in a more fragile state. Mothers described being motived by the immunological benefits of breastfeeding and explained that the pandemic and associated mitigation strategies changed the infant feeding context. Several mothers discussed how their time demands changed, so they could devote more time to caring for their babies with fewer work demands. The qualitative analysis of this study also showed that in this new pandemic context of the postpartum period, that spousal support was important, and spouses provided emotional and tangible support to postpartum mothers. On the other hand, the social distancing for nuclear families provided mothers the opportunity to try novel mothering styles because of the reduced the influence of older generations on infant feeding [56, 57]. Mothers who exclusively breastfeed felt that the mitigation strategies had positive effects on their breastfeeding practices because they had more time to breastfeed, recognized the economic benefits, got more support from their spouse, and some of the typical social obstacles were removed.

The results of this study helped contextualize the impact of the COVID-19 pandemic on infant feeding practice. It can be used to guide the implementation of programming that supports optimal infant feeding practices beginning in the antenatal period since intention remains a powerful factor in actual practice. Socially distanced face-to-face or telehealth visits should include positive breastfeeding messaging with emphasis on how breastfeeding supports infant health and boosts immunity and can reduce expenses. Having socially distanced face-to-face or telehealth discussions about long-term infant feeding plans with mothers and their spouses before discharge offers another intervention node on the pathway to infant feeding decisions. Moreover, health providers must be sensitive to the important concerns surrounding the COVID-19 situation in relation to parent roles, so they can convey that breastfeeding can be done safely. Given the importance of socioeconomic status, work status, maternal age, social network factors, lactation consultants tailored consultations are especially crucial as the economic and social changes on family dynamics continue to play out. While household socioeconomic hardship may pressure family members to look for ways to earn more income, the cost savings associated with breastfeeding can be considered in this equation.

## Conclusions

Mothers with higher socioeconomic status and who unemployed or worked from home were more likely to exclusively breastfeed during the second wave of the COVID-19 outbreak in Thailand. Healthcare providers can supply tailored programs for breastfeeding mothers, based on awareness of socioeconomic status, work status, maternal age, and social network. Furthermore, optimal exclusive breastfeeding outcomes will be improved through supportive resources, intention programs, and spouse involvement, particularly for women who face financial difficulties and work outside the home during a pandemic.

## Data Availability

The dataset or transcripts are available from the corresponding author upon reasonable request.
